# Highly variable effect of sonication to dislodge biofilm-embedded *Staphylococcus epidermidis* directly quantified by epifluorescence microscopy: an in vitro model study

**DOI:** 10.1186/s13018-020-02052-3

**Published:** 2020-11-11

**Authors:** Erik T. Sandbakken, Eivind Witsø, Bjørnar Sporsheim, Kjartan W. Egeberg, Olav A. Foss, Linh Hoang, Geir Bjerkan, Kirsti Løseth, Kåre Bergh

**Affiliations:** 1 Department of Orthopedic surgery, St Olav’s University Hospital, Trondheim, Norway; 2 Cellular and Molecular Imaging Core Facility (CMIC), Norwegian University of Science and Technology, Trondheim, Norway; 3 Neuromedicine and Movement Science (INB), Norwegian University of Science and Technology, Trondheim, Norway; 4 Department of Clinical and Molecular Medicine, Norwegian University of Science and Technology, Trondheim, Norway; 5 Department of Medical microbiology, St Olav’s University Hospital, Trondheim, Norway

**Keywords:** Sonication, Biofilm formation, *Staphylococcus epidermidis*, Fluorescence microscopy, Confocal microscopy, Electron microscopy

## Abstract

**Background:**

In cases of prosthetic joint infections, culture of sonication fluid can supplement culture of harvested tissue samples for correct microbial diagnosis. However, discrepant results regarding the increased sensitivity of sonication have been reported in several studies. To what degree bacteria embedded in biofilm are dislodged during the sonication process has to our knowledge not been fully elucidated. In the present in vitro study, we have evaluated the effect of sonication as a method to dislodge biofilm by quantitative microscopy.

**Methods:**

We used a standard biofilm method to cover small steel plates with biofilm forming *Staphylococcus epidermidis* ATCC 35984 and carried out the sonication procedure according to clinical practice. By comparing area covered with biofilm before and after sonication with epifluorescence microscopy, the effect of sonication on biofilm removal was quantified. Two series of experiments were made, one with 24-h biofilm formation and another with 72-h biofilm formation.

Confocal laser scanning microscopy (CLSM) and scanning electron microscopy (SEM) were used to confirm whether bacteria were present after sonication. In addition, quantitative bacteriology of sonication fluid was performed.

**Results:**

Epifluorescence microscopy enabled visualization of biofilm before and after sonication. CLSM and SEM confirmed coccoid cells on the surface after sonication. Biofilm was dislodged in a highly variable manner.

**Conclusion:**

There is an unexpected high variation seen in the ability of sonication to dislodge biofilm-embedded *S. epidermidis* in this in vitro model.

## Background

Development of prosthetic joint surgery is one of the major successes in medicine over the last century. Prosthetic joint infection (PJI) is a devastating complication after prosthetic surgery. Despite efforts to minimize the rate of PJI, it is an increasing problem [7, 14]. A major challenge is the evolving bacterial resistance to antibiotics and bacteria forming biofilm which makes it difficult to treat PJI.

Culture of harvested tissue biopsies during prosthetic revision is commonly used when diagnosing a PJI. The sensitivity of bacterial culture of tissue samples is not optimal. The reported sensitivity based on standardized criteria and rigorous tissue sampling technique differs between 86 and 89% [10, 16, 17]. It has been claimed that sonication of explanted prostheses with subsequent culture of sonication fluid can increase the sensitivity of the test compared to culture of tissue samples [16, 18, 25, 27].

However, these results have not been unambiguously reproducible. This is apparent in the ongoing debate concerning sonication [8, 29], and sonication findings are not included in the Musculoskeletal Infection Society (MSIS) criteria for periprosthetic infection. First, in vitro studies raise the question whether *Staphylococcus epidermidis* biofilm might respond to sonication unlike other species [4, 5, 19]. Second, clinical studies, including refinement with PCR-techniques, show no difference in sensitivity of culture of tissue samples compared to culture of sonication fluid [1, 12]. Finally, lower sensitivity of culture of sonication fluid compared to culture of standard tissue samples have been reported [5, 6, 9, 10, 28].

The abovementioned observations raise the question whether sonication, as a method to dislodge biofilm in general, is as effective as often claimed. Studies have focused on detachment of biofilm bacteria, and to the best of our knowledge, we are not aware of any microscopic studies describing potentially remaining biofilm on a metal surface after sonication. Epifluorescence microscopy offers the opportunity to directly quantify the amount of bacteria on a surface by measuring the area covered by biofilm [13, 20]. We here present a method where epifluorescence is employed to visualize the area covered by a biofilm before and after sonication. Our aim was to evaluate the capability of the model to quantify the effect of sonication as a method to dislodge biofilm embedded *S. epidermidis* from the surface of steel plates, in vitro.

## Methods

### Methodological overview

**Fig. 1:**
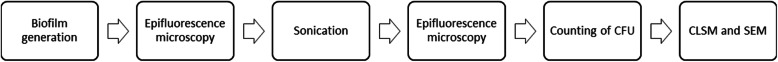
A flowchart of the experimental design describes the steps performed with 24-h biofilm. 46 steel plates with established 24-h biofilm were subjected to epifluorescence microscopy before and after sonication. The number of CFU in the sonication fluid was calculated. Confocal laser scanning microscopy (CLSM) was applied to 4 of the 46 specimen and scanning electron microscopy (SEM) to 2 of the 46 specimen for confirmation of possible presence of coccoid bacteria after sonication. 2 additional positive controls, which were not sonicated, were visualized with SEM to see that the preparation did not affect the biofilm

We used a standard biofilm procedure to cover steel plates with biofilm and carried out the sonication procedure according to clinical practice [5, 27]. By comparing area covered with biofilm before and after sonication, the effect of sonication on biofilm removal was quantified. Two series of experiments were made, the first with 24-h biofilm formation and the second with 72-h biofilm formation. A schematic overview is presented in Fig. 1.

Confocal laser scanning microscopy (CLSM) and scanning electron microscopy (SEM) were used to confirm whether bacteria were present after sonication. In addition, counting of colony-forming units (CFU) before and after sonication served as a measure of the effect of sonication on dislodging viable bacteria.

### Bacterial strains, inoculum, and culture conditions

*Staphylococcus epidermidis* ATCC 35984 (American Type Culture Collection) was stored in glycerol at − 80 °C and after thawing spread onto blood agar plates and incubated in ambient air at 37 °C overnight. Colonies were harvested from blood agar and suspended in tryptic soy broth with 1% glucose (TSB-Glu) to a turbidity of 0.5 McFarland equivalent to approximately 1.5 × 10^8^ colony-forming units (CFU)/mL. To document the initial concentration of the inoculum, quantitative cultures were performed. The capacity of *S. epidermidis* ATCC 35984 for biofilm generation was demonstrated by a modification of the original method as described elsewhere [23, 24].

### Biofilm formation on steel plates and specimen preparation

In the first experiment, 46 sterilized quadratic steel plates and 6 additional controls (~ 24 mm^2^ AISI 316L RA, surface roughness 0.06–0.08 μm, Skala Fabrikk, Terminalen 6, N-7080 Heimdal, Norway) were placed singly into wells of Nunc 6-well cell culture plate (Thermo Scientific, https://www.thermofisher.com) containing 3 mL of TSB-Glu and *S. epidermidis* ATCC 35984 1.5 × 10^8^ CFU/mL. The steel plates were handled with the biofilm side facing upwards to avoid mechanical disruption throughout the experiment. Incubation was performed in ambient air at 37 °C for 24 h without stirring [13]. After incubation, each plate was rinsed 3 times in separate wells (Nunc 24-well cell culture plate) containing 2-mL sterile saline and gently vortexed at 400 rpm for 10 s. One-milliliter sterile saline was gently poured over the plate from a pipette during transfer to the next well to minimize carry-over of planktonic bacteria. The concentration of bacteria in the final rinsing fluid served as a baseline for evaluating the effect of sonication.

Four negative controls were processed parallel to specimens, of which 2 were incubated solely in TSB-Glu before epifluorescence microscopy and 2 underwent microscopy directly from the sterile packaging. All negative controls were stained with Live/Dead™ BacLight ™ Bacterial Viability Kit (Thermo Fischer Scientific, L7012) before microscopy. Two positive controls were processed parallel to specimens and served as controls for the SEM preparation procedure.

The second experiment with 12 plates and 2 additional negative controls (~ 23 mm^2^) was carried out with equal setup as for the 24-h experiment. The time of biofilm growth was extended to 72 h with exchange of TSB-Glu nutrition every 24 h. The 2 negative controls were processed parallel to specimens, one incubated solely in TSB-Glu and the other underwent microscopy directly from the sterile packaging.

### Sonication of steel plates

A BactoSonic® sonicator (Bandelin electronic GmbH & Co. KG) was operated according to the manufacturer’s operating instructions. The bath was filled 2/3 with water and 95 ml Tichopur TR3 added before degassing at maximum effect for 15 min. The overall efficacy was controlled with the “foil test” followed by a detailed evaluation using a Bruel & Kjær 8103 hydrophone (see Additional file 1 for description).

**Fig. 2:**
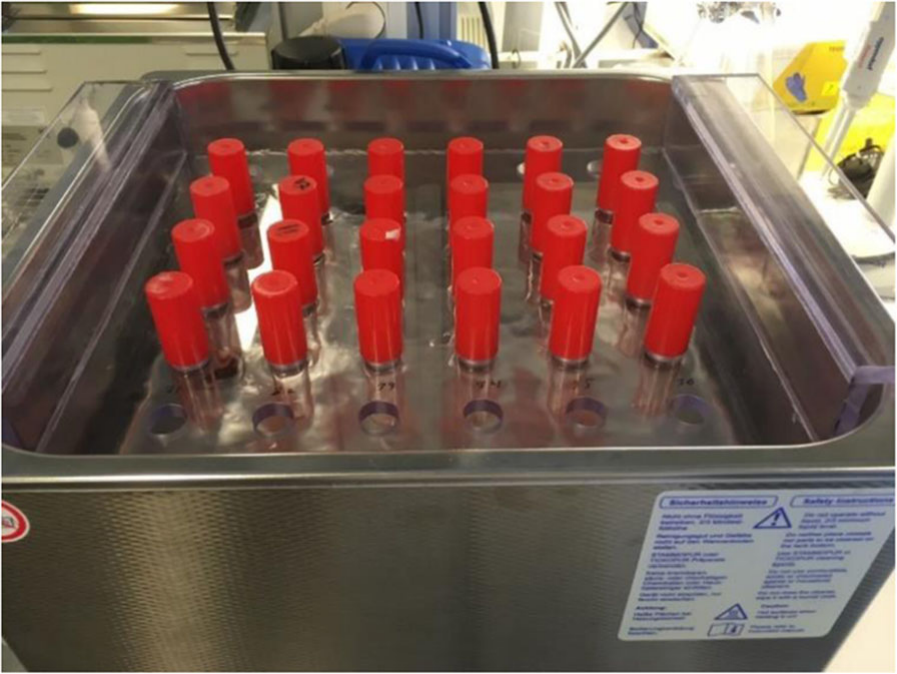
Steel plates were sonicated in standard glass test tubes in a customized stand for correct and standardized positioning in the bath

The test tubes containing 10-mL sterile saline and one steel plate with the investigated surface facing upwards were sonicated at 100% effect (800 W) for 5 min at room temperature (Fig. 2). The sonication fluid was then aspirated and transferred to sterile bottles before serial dilution and inoculation on blood agar plates.

### Quantification of bacteria before and after sonication

Ten microliters of undiluted final rinsing fluid and 10 μL of sonication fluid diluted 1:10 and 1:100 were seeded onto blood agar plates for counting of CFU. Pilot studies (data not shown) demonstrated adequate removal of planktonic bacteria during prior rinsing steps.

### Staining and visualization of bacteria with epifluorescence microscopy before and after sonication

Staining of the biofilm was obtained with LIVE/DEAD ™ BacLight ™ Bacterial Viability Kit (Thermo Fischer Scientific, L7012) according to the manufacturer’s protocol. The plates were placed with the surface to be investigated facing downwards onto the object glass with an integrated coordinate system (Ibidi μ-Slide 8 Well Grid 500, uncoated).

An inverted EVOS™ FL Auto 2 Imaging System enabled visualization of the entire surface. Gain and time of exposure were adjusted to avoid picture saturation and kept constant throughout the experiment. Staining was repeated and imaging performed with identical settings after the sonication procedure to visualize remaining bacteria.

### Staining and visualization of bacteria with CLSM after sonication

Two sonicated plates from the 24-h experiment and one plate from the 72-h experiment, with remaining biofilm as demonstrated with epifluorescence, were subjected to CLSM. An inverse confocal laser scanning microscope LSM510 (Carl Zeiss AG) equipped with a C-Apochromate 63x/1.2 water objective was used for confirmation of biofilm embedded bacteria. The Cyto 9 dye and propidium iodine component of the viability kit was excited by 488-nm laser line from a 30-mW Argon laser and detected using the filters BP 505–530 nm and LP 615 filter, respectively. One Airy unit was used for both channels to keep high signal-to-noise ratio. Z-stacks of biofilm data were rendered as 3D-images with Imaris-Microscopy Image Analysis Software, Oxford Instruments (version 8.2.1).

### Preparation for SEM after sonication

Two of the 46 sonicated plates with remaining biofilm and 2 positive controls were fixed with a solution of 2.5% glutaraldehyde with 2% paraformaldehyde and 0.075% Ruthenium Red in 0.1 M Hepes buffer for 4 h at room temperature, washed in 0.1 M Hepes buffer, and subsequently dehydrated using increasing ethanol concentrations (10, 25, 50, 70, 90, 2 × 100%), for 5–10 min each, followed by drying using hexamethyldisiloxane (HMDS) (50% diluted with ethanol and 2 × 100%), for 20 min each and transferred to a desiccator to avoid water contamination. After drying, the samples were mounted on aluminum pin with double-sided carbon tape and sputter coated (Leica ACE600) with 30 nm gold/palladium. Samples were examined using a scanning electron microscope (VolumeScope SEM, Thermo Fischer Scientific) at a voltage of 7 kV.

### Quantification by measurement of area covered by biofilm

**Fig. 3:**
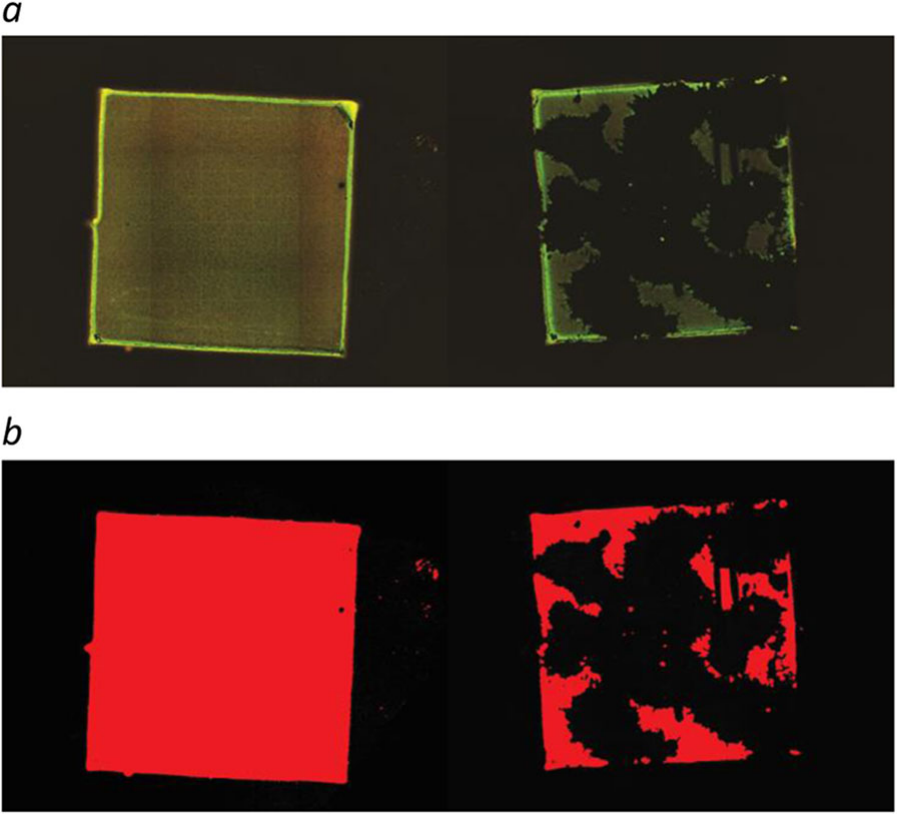
The effect of sonication is seen by comparing area covered by biofilm before and after sonication visualized by epifluorescence (**a**). To be able to quantify the covered area, pictures were dichotomized with help of a macro in the Fiji software (**b**). The resulting red area represents biofilm

Epifluorescence pictures were processed with Fiji [21] using a custom macro. The macro calibrates the pixel size, mean filters (10 px radius), and automatically sets a threshold based on LI-algorithm (LI dark) and measures the segmented area. By this, a gradient picturizing of the biofilm was transformed into a dichotomized picture and thereby making the quantification of the biofilm more reliable (Fig. 3). The area covered by biofilm before and after sonication was expressed as mm^2^.

### Statistics

Statistical analyses were performed using the software package IBM SPSS Statistics for Windows, Version 23.0. Armonk, NY: IBM Corp. Biofilm-covered area is presented in boxplots. Otherwise, data are presented as median and range.

Correlation between area covered by biofilm after sonication and the corresponding CFU in the sonication fluid is presented as scatter plots. The Spearman rank correlation is used to describe the correlation between area covered by biofilm after sonication and the corresponding CFU in the sonication fluid.

## Results

### Quantitative effect of sonication on biofilm removal

**Fig. 4:**
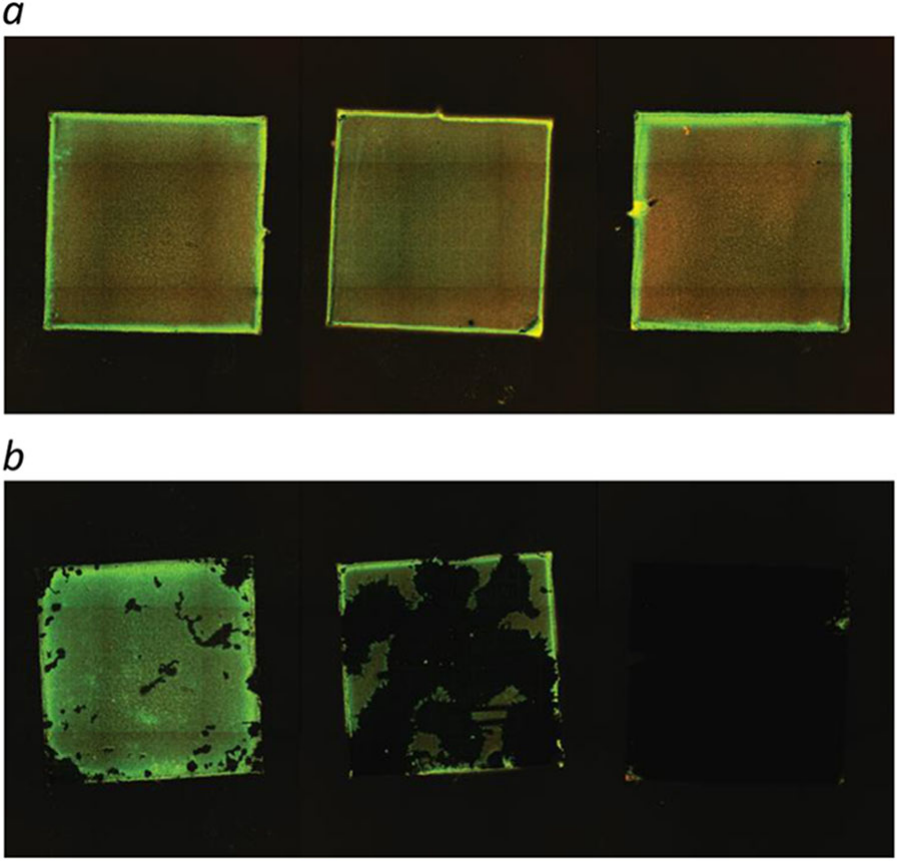
The figure illustrates variation seen in area covered by biofilm after sonication. Epifluorescence images show establishment of biofilm after 24-h incubation (before sonication (**a**), after sonication (**b**))

In the 24-h experiment, biofilm was established in a uniform manner covering the entire surface on all 46 plates investigated by epifluorescence microscopy. Sonication of the plates yielded highly variable results with respect to the capability of dislodging biofilm from the surface (Table 1 and Fig. 4). No formation of biofilm appeared on 4 negative controls.

**Table 1:** The table shows area covered by biofilm on the steel plates before and after sonication in both experiments (12-h and 72-h biofil

		Median (mm^2^)	25–75% percentile (mm^2^)	Minimum Maximum (mm^2^)
12-h biofilm (46 plates)	Before sonication	25.3	25.1–25.6	23.3–26.7
	After sonication	1.1	0.4–6.8	0.0–22.2
72-h biofilm (12 plates)	Before sonication	28.3	27.9–29.3	27.5–30.0
	After sonication	22.0	0.1–28.8	0.1–30.7

In the 72-h experiment, epifluorescence microscopy showed that biofilm was established in a uniform manner on all 12 plates. Compared to the 24-h biofilm experiment, sonication resulted in less pronounced dislodgment of biofilm (Table 1). No biofilm formation appeared on 2 negative controls.

### Visualization of biofilm bacteria after sonication

**Fig. 5:**
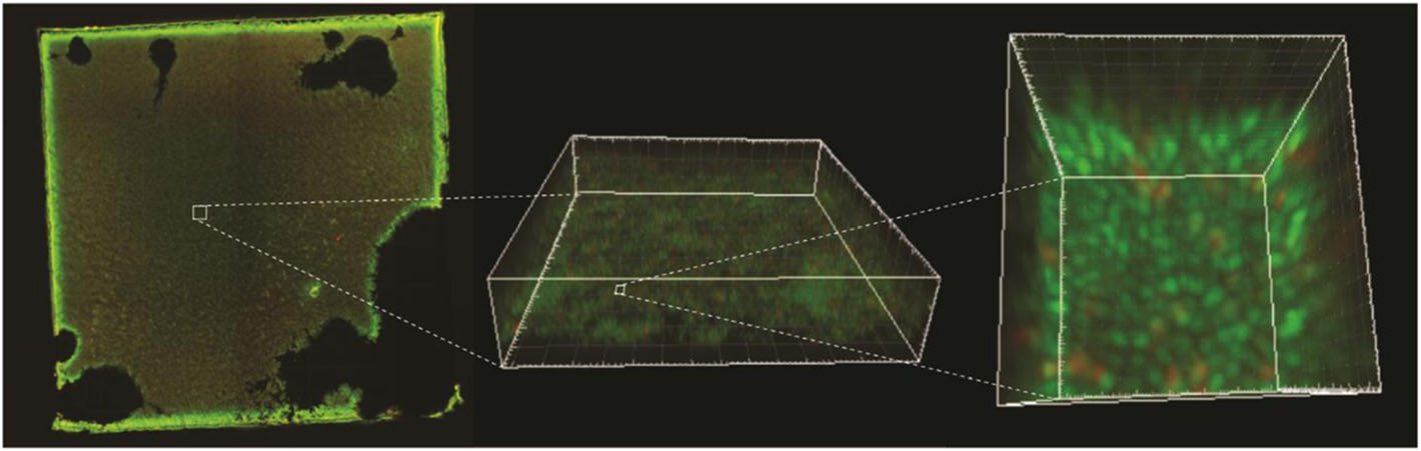
Epifluorescence image of a plate after sonication (a). A marked section (stippled line) of the epifluorescence image is visualized with confocal laser scanning microscopy where multiple z-stacks are rendered as a 3D-image (b). A section of this image is further magnified and rendered as a 3D-image with a different viewing angle (c). Coccoid bacteria are evident as green (live) and red (dead) cells

The presence of biofilm-embedded bacteria after sonication was confirmed by CLSM (Fig. 5).

**Fig. 6:**
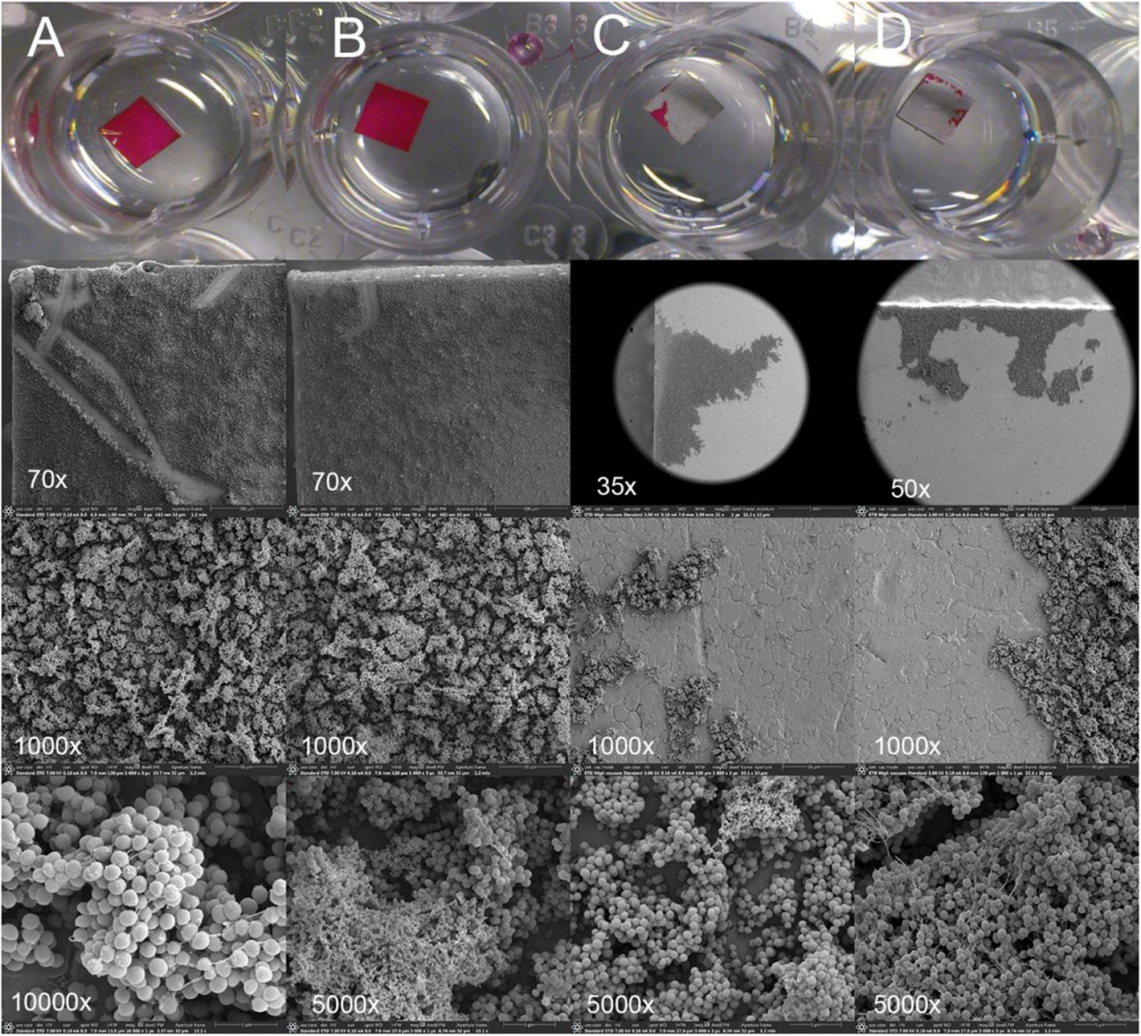
Two positive control steel plates (A and B, not sonicated) covered with biofilm processed for SEM and photographed with increasing magnification. Plate A (× 70 magnification) shows a scratch from the forceps used for handling indicating a fragile binding to the surface. 2 of the 46 plates (C and D) were processed for SEM after sonication

Remaining coccoid bacteria in cluster-like formations were evident on the 2 steel plates with 24-h biofilm selected for SEM after sonication (Fig. 6).

### Culture of rinsing and sonicate fluid

Culture of final rinsing fluid and sonicate fluid was positive in all samples (46 + 12). In the 24-h biofilm the amount was 2 × 10^3^ (5 × 10^1^–5 × 10^4^) and 9 × 10^4^ (6 × 10^4^–3 × 10^5^) CFU/mL, respectively. In the 72-h biofilm, the amount was 1 × 10^4^ (6 × 10^3^–3 × 10^4^) and 8 × 10^5^ (1 × 10^5^–2 × 10^6^) CFU/mL, respectively.

**Fig. 7:**
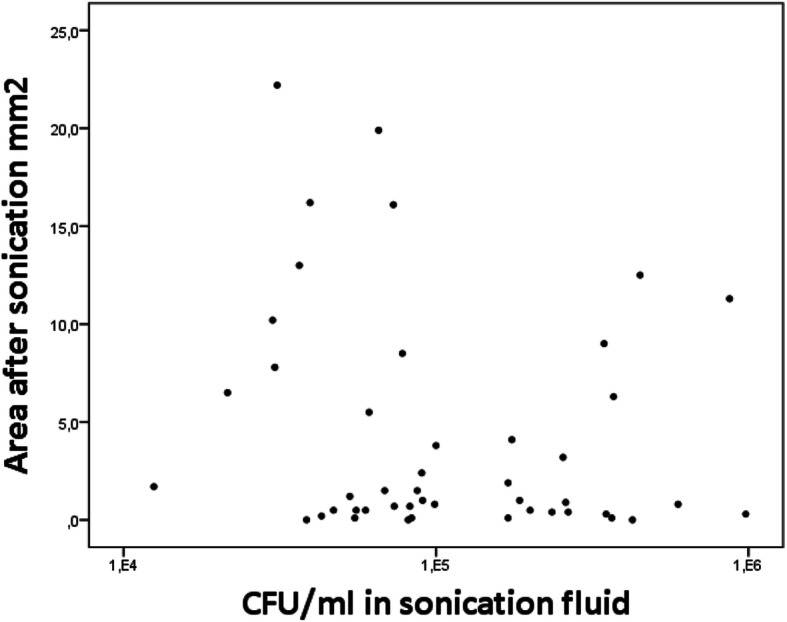
Scatter plot of 24-h biofilm. The amount of remaining bacteria after sonication (covered area) on the steel plates is plotted against the number of CFU in the sonication fluid

**Fig. 8:**
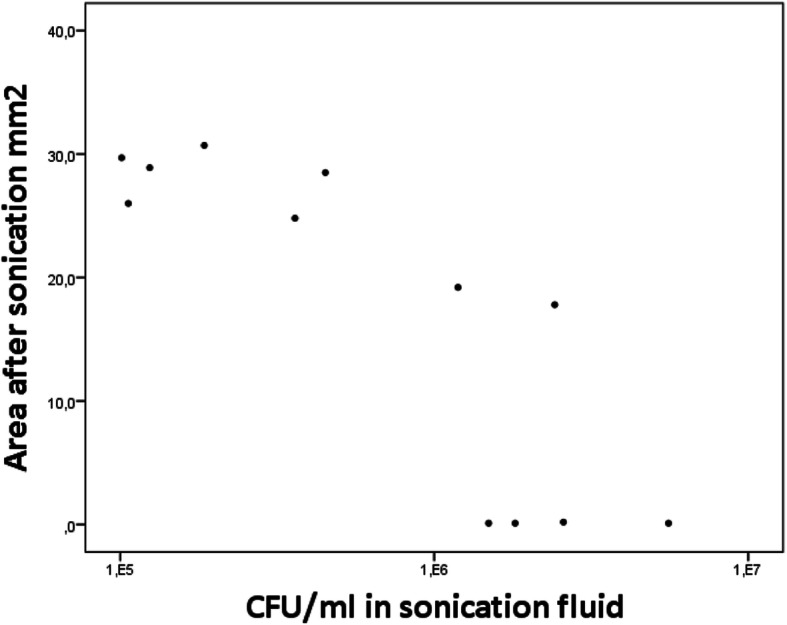
Scatter plot of 72-h biofilm. The amount of remaining bacteria after sonication (covered area) on the steel plates is plotted against the number of CFU in the sonication fluid

The amount of bacteria in the sonication fluid and the corresponding area covered by biofilm after sonication is presented in the scatter plots (Figs. 7 and 8). The correlation coefficient was − 0.213 and − 0.838 for the 24-h and 72-h biofilm, respectively.

## Discussion

In this study, we established a method to quantify the effect of sonication as a method to dislodge biofilm-embedded *S. epidermidis* from a steel surface in vitro. Our study stands out compared to existing literature by showing biofilm changes on a large surface directly by quantitative microscopy and adds new knowledge about how biofilm responds to a clinically relevant sonication protocol. The main result is the highly variable manner by which sonication resulted in dislodgment of biofilm as visualized by epifluorescence and SEM. After ensuring an even effect of ultrasound inside all tubes at all positions in the bath, we believe the variability observed can be trusted and not be attributed to technical issues. We used a stepwise approach to quantify biofilm-covered area by employing 3 techniques of microscopy. Epifluorescence microscopy enabled us to visualize total plate areas before and after sonication while confocal laser scanning microscopy and scanning electron microscopy verified bacterial growth on the plates. Given the biofilm pattern that was often scattered across the surface, one could easily make the mistake of depicting an area not covered by biofilm when biofilm in fact covered a large portion of the surface. Avoidance of fixation was crucial, as we wanted to observe the quantitative changes on every plate before and after sonication with subsequent counting of CFU from the sonication fluid from the same plates. We found it advantageous to use an in vitro model simple for others to reproduce, but acknowledge that in vivo models would have been advantageous for achieving more clinically relevant results.

The variable effect of sonication on biofilm removal presented here is in part contradictory to other reports from in vitro experiments claiming that sonication alone completely dislodges biofilm [2] or in combination with autoclaving [15]. Supporting findings exist where biofilm embedded *S. epidermidis* did not dislodge completely [22]. Biofilm could be detected microscopically by qualitative analysis after sonication, but the sonication was performed with a handheld probe meant for operative use and therefore not directly comparable to our results. The methodological description is often scant for numerous in vitro studies using sonication as a means to dislodge biofilm for subsequent quantification. Our results show larger variation after sonication in the more mature 72-h biofilm compared to the 24-h biofilm, but this only applies to our in vitro model. When carrying out an in vitro experiment, one should consider if biofilm might still adhere to the object after sonication. This could lead to unreliable results when doing subsequent quantification of dislodged bacteria.

Several clinical trials have not convincingly proved sonication as a superior method to recover viable bacteria in cases of PJI. One might speculate whether the variation seen in our study also applies to in vivo biofilms in chronically infected prostheses, and thus explain why culture of sonication fluid has not been unanimously reported superior to culture of tissue samples. The differing results in the literature regarding the sensitivity of culture of tissue samples compared to cultures of sonication fluid could be linked to our results showing a highly variable effect of sonication. It might be that in vivo biofilms are even more resistant to sonication than in vitro biofilms. One should consider inadequate removal of biofilm during sonication as a reason for lower sensitivity for sonication fluid compared to tissue samples, as problematized in the introduction [5, 6, 9, 10, 28]. CFU counting of dislodged bacteria is a standard method for quantification. One must assume uncertainties in CFU results since biofilm dislodge in aggregates [11, 26]. We chose not to include vigorous vortex-mixing in conjunction with sonication in the protocol as it would be impossible to distinguish whether biofilm detachment was due to vortex-mixing or sonication. Additional vortex-mixing is employed in some clinical studies, and we acknowledge that this might increase the efficacy of the protocol. Compared to final rinsing fluid, we observed a considerable increase in CFU/mL after sonication in all samples. There was a 10-fold higher number of CFU in the sonication fluid from the 72-h experiment compared to the 24-h experiment. This is most likely due to the prolonged time of biofilm growth resulting in more biofilm mass [3]. It is possible that the correlation between area covered by biofilm after sonication and the increased number of bacteria in the sonication fluid is detectable because of increased bacterial mass after 72 h of incubation. The change of nutrient every 24 h was essential to facilitate maturation of the biofilm while preserving bacterial viability.

Our study is limited because generalization of the results into clinical conditions is problematic and hampered with uncertainties. Further studies should include in vivo biofilms. The highly variable effect of sonication seen in our experiments only applies to in vitro biofilm established under simple static culture conditions. Results from the 72-h experiment must be interpreted with care as the number of specimens is low. However, we do not suspect less variation by increasing the number of specimen. Results are also limited to one *S. epidermidis* strain, although this species is the most frequent pathogen in PJI.

Alternative methods for quantification might be measurement of optical density from resolved biofilm on the plates after sonication, or measurement of heat production from dislodged biofilm by microcaloritmetry [15, 19].

Changes solely in biofilm thickness, rather than area covered by biofilm, would not be detected with our procedure, and it is important to emphasize that measurement of area covered by biofilm does not take into account the variable thickness of the biofilm. To better elucidate this issue, we considered measurement of relative fluorescence instead of area covered by biofilm. However, this involves an extensive standardization of image acquisition, which in our opinion implies greater limitations and uncertainties.

## Conclusion

This in vitro study shows a highly variable effect of sonication to dislodge biofilm-embedded *Staphylococcus epidermidis* as quantified directly by epifluorescence microscopy.

## Supplementary Information

**Additional file 1:.** Quantification of ultrasound distribution in the ultrasonic bath.

## Data Availability

All data generated or analyzed during this study are included in this published article and its additional file.

## Abbreviations

PJI: Prosthetic joint infection
CLSM: Confocal laser scanning microscopy
SEM: Scanning electron microscopy
ATCC: American Type Culture Collection
TSB-Glu: Tryptic soy broth with 1% glucose
CFU: Colony-forming units
