# Supplementary material for: Highly variable effect of sonication to dislodge biofilm-embedded Staphylococcus epidermidis directly quantified by epifluorescence microscopy: an in vitro model study
Source: J Orthop Surg Res. 2020 Nov 11;15:522. doi: 10.1186/s13018-020-02052-3 (PMC7661210; doi:10.1186/s13018-020-02052-3)
Supplement: Supplementary file 1 — Additional file 1:. Quantification of ultrasound distribution in the ultrasonic bath. [file 13018_2020_2052_MOESM1_ESM.docx]

**Appendix: Quantification of ultrasound distribution in the ultrasonic bath**

The BactoSonic® sonicator (Bandelin electronic GmbH & Co. KG) was prepared according to manufacturer’s instructions. The bath was filled with water and preparation (Tichopur TR3) up to the fill level mark, and the solution degassed for 15 minutes with the sonicator set to maximum power. Standard glass laboratory test tubes were filled with 7 ml 0,9% NaCl solution and placed in a custom-made test tube stand fabricated to standardize the position of 24 test tubes (figure 2). A hydrophone (Brüel & Kjær, Nærum, Denmark, type 8103) was placed inside the test tube. The tip of the hydrophone was positioned 54 mm above the bottom of the sonication bath resembling the same position as the metal plates to be sonicated. The signal from the hydrophone was recorded at each tube position. Data were recorded using Picoscope 2203 and software Picoscope 6 (Pico® Technology Limited). 5 repeated measurements of 50ms were recorded at each of the 24 positions. The sampling frequency was 156 kHz. The fundamental sonication frequency was estimated to 38.5 KHz. Second harmonics were included when estimating signal power. The hydrophone was characterized with a flat frequency response ranging from 0.1Hz to 180 kHz, indicating equal sensitivity for all measured frequencies. The hydrophone directivity pattern was also approximately circular for the measured frequency range, so orientation of the probe should not affect the measurements. The ultrasound power at different hole-positions within the tank was therefore estimated by using the RMS-voltage of the hydrophone signal (figure 1). The average (SD) voltage for all positions was 1.64V (0.42V).

*Figure 1 to appendix:*

*Figure 2 to appendix*

**Figure and legends to appendix**

*Figure 1: Error bar plot showing average RMS voltage and 95% Ci of the mean for each of the 24 tubes based on 5 repeated measures. Dotted lines represent the mean value (1.64V) for all measurements.*


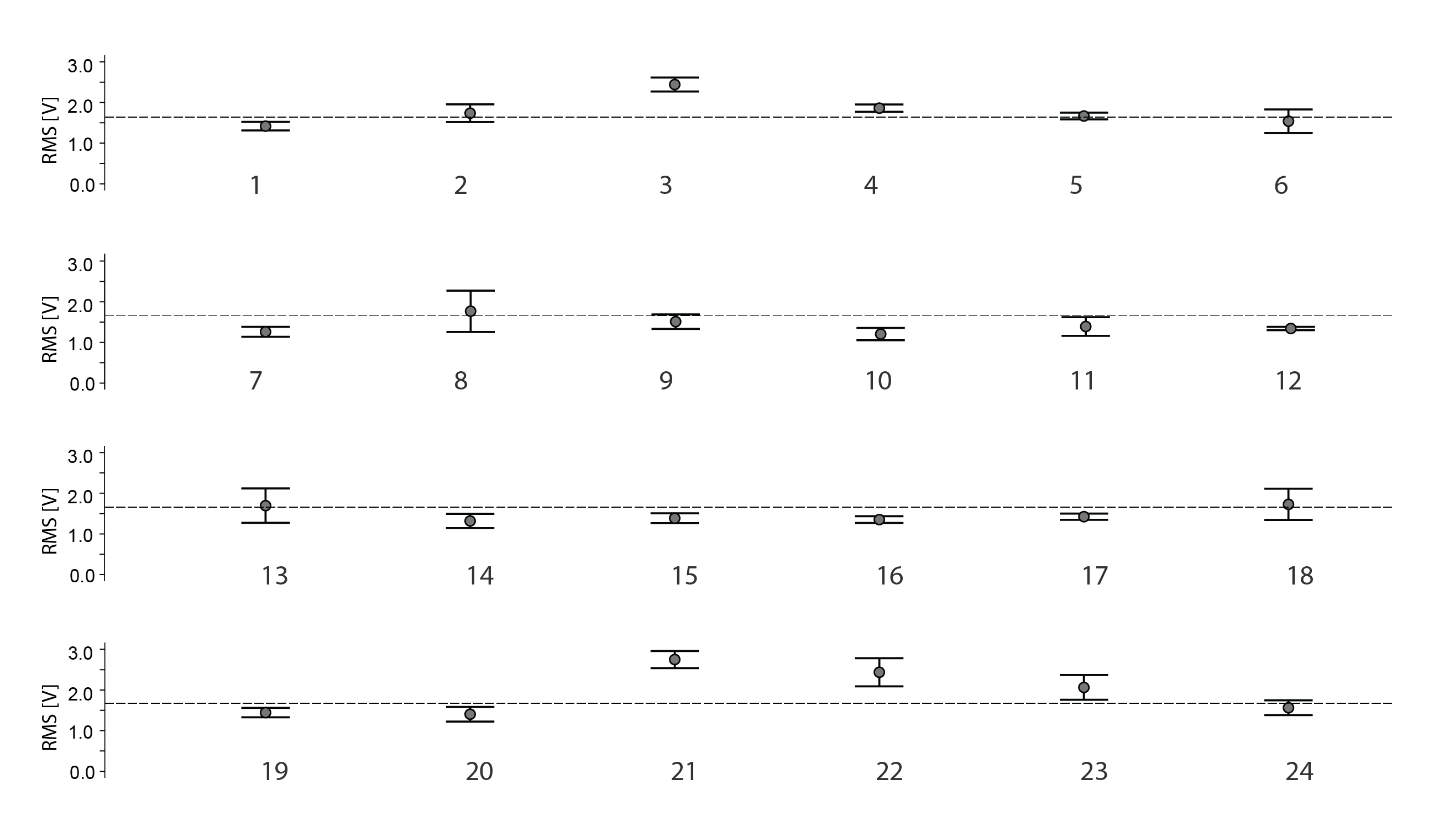


*Figure 2:*
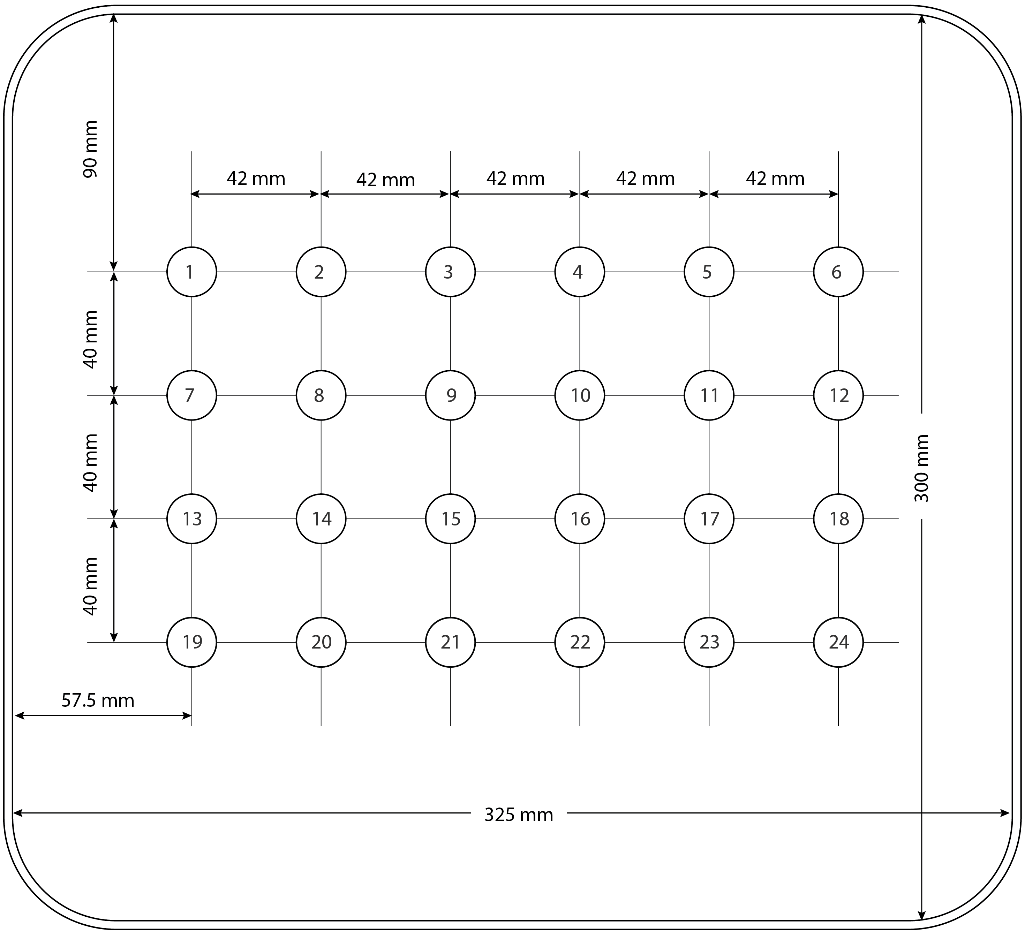
*Tube positions relative to the sonication tank.*
